# Use of anchorchip-time-of-flight spectrometry technology to screen tumor biomarker proteins in serum for small cell lung cancer

**DOI:** 10.1186/1746-1596-5-60

**Published:** 2010-09-20

**Authors:** Jie Du, Shuanying Yang, Xiuli Lin, Lina Bu, Yandong Nan, Shufen Huo, Wenli Shang

**Affiliations:** 1 Department of Respiratory Medicine, Second Affiliated Hospital of Medical School, Xi'an Jiaotong University, N-710000 Xi'an, China; 2 Medical Department of Second Hospital of Xi'an, N-710000 Xi'an, China

## Abstract

**Background:**

The purpose of this study is to discover potential biomarkers in serum for the detection of small cell lung cancer (SCLC).

**Methods:**

74 serum samples including 30 from SCLC patients and 44 from healthy controls were analyzed using ClinProt system combined with matrix-assisted laser desorption/ionization time-of-flight masss spectrometry (MALDI-TOF-MS). ClinProt software and genetic algorithm analysis selected a panel of serum markers that most efficiently predicted which patients had SCLC.

**Results:**

The diagnostic pattern combined with 5 potential biomarkers could differentiate SCLC patients from healthy persons, with a sensitivity of 90%, specificity of 97.73%. Remarkably, 88.89% of stage I/II patients were accurately assigned to SCLC.

**Conclusions:**

Anchorchip-time-of-flight spectrometry technology will provide a highly accurate approach for discovering new biomarkers for the detection of SCLC.

## Background

Early diagnosis appears to be the most appropriate tool to reduce disease-related mortality. With the advent of proteomics, the comparison of large numbers of proteins in complex biological samples such as serum has become feasible. Recently, new strategies that facilitate proteomic analysis by magnetic beads dramatically simplifying the preanalytical sample separation and coupling with mass spectrometry (MS) have been introduced for biomarker discovery research. The matrix-assisted laser desorption/ionization time-of-flight masss spectrometry (MALDI-TOF MS) profiling has been successfully used to differentiate colorectal l[1], breast, prostate[2], and bladder cancer from controls. Similar studies of lung cancer have not been reported yet.

In this study, we analyzed serum samples from SCLC patients and healthy individuals using ClinProt system. We could find potential biomarkers in SCLC and establishing the pattern for discriminating SCLC patients from healthy controls.

## Materials and methods

### Cancer patients and controls

Serum samples including 30 SCLC patients and 44 healthy individuals were obtained from the serum banks of the Department of Respiratory Medicine, Second Affiliated Hospital of Medical School of Xi'an Jiaotong University from October 2003 to May 2008. SCLC group had a median age of 51.68 years(ranging from 33 to71 years, 25 men and 5 women) and consisted of 9 stage I/II and 21 stage III/IV patients according to the International Union Against Cancer (UICC)staging system of lung cancer. Diagnoses were pathologically confirmed, and specimens were obtained before treatment. The median age of the control group with no evidence of disease was 49.0 (ranging from 44 to76 years, 28 men and 16 women). All serum samples were separated by centrifugation, then immediately aliquoted and stored in a dedicated -80°C freezer. Approval for the study was given by the Regional Ethical Committee.

### Isolation of peptides

Peptides were captured and concentrated using magnetic beads based weak cation exchange (MB-WCX) on the ClinProt robotic platform (Bruker Daltonics, Billerica, MA) according to the manufacturer's specifications. All analyses were performed in a 96-well format using the same batch of magnetic particles. This system automates all liquid handling steps, including magnetic separation via a robotic manipulating arm, mixing of eluates with MALDI matrix, and deposition onto the Bruker 384-spot MALDI target plates.

### MALDI Analysis

Peptide profiles were analyzed with an Autoflex MALDI-TOF mass spectrometer (Bruker, Billerica, MA) as described [3]. Separate spectra were obtained for the restricted m/z ranges, corresponding to polypeptides with molecular mass of 800-40000 Da under specifically optimized instrument settings. Each spectrum was the result of 400 laser shots. Peptide samples were always mixed with 10 μL premade a-cyano-4-hydroxycinnamic acid (ACCA) matrix solution (Agilent), deposited onto the stainless steel target surface in every other column of the 384-spot layout, and allowed to dry at room temperature. A weekly performance test using commercial human reference serum (Sigma catalog number S-7023, lot 034K8937) was done and the experiment was duplicated in exactly same order. Hereafter, the entire process of capturing and concentrating serum proteins using magnetic beads including the generation of readouts of the MALDI-TOF spectra will be designated as the protein profiling procedure.

### Bioinformatics analysis

A k-nearest neighbor genetic algorithm contained in the software suite was used to identify statistically significant differences in protein peaks in the groups analyzed. The peaks inputted to the model with highest accuracy were selected as the set of potential biomarkers. After the model was generated, a 20% leave out cross-validation process was performed within the software. Only the cross-validated values were used for the reported classifications. The peaks were filtered to maintain a S/N of more than three. The protein fingerprint data were analyzed by FlexAnalysis3.0. Comparisons between SCLC group and control group were performed with the Wilcoxon test. Statistical significance was assumed when *P *was < 0.05.

## Results

### Peptide Profiling of SCLC patient Sera

**Figure 1:**
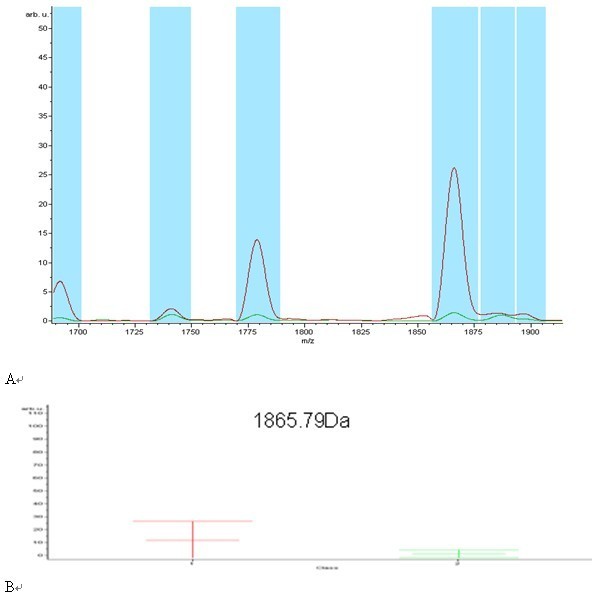
**Representative MALDI-TOF MS serum spectra(1.7-1.9 kD)**. (A)Two differentially expressed proteins of m/z 1778.67 and 1865.79 Da were screened up; (B) It showed the bar graphs, the mean values and the standard deviations of the log-normalized intensities for the SCLC subjects (red) and the healthy individuals (green).

**Figure 2:**
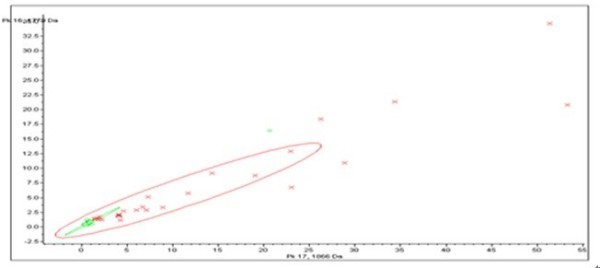
**Scatter plot of the top two peaks on basis of which the classification patient-control group was made: The x-axis represented 1865.79 Da and the y-axis represented 1778.67 Da in order to observe their ability of sample distribution**. The farrago area was small and it showed that they could distinguish SCLC (red) from controls (green) effectively.

System reproducibility was verified on the same day by visual comparison of 13 reference samples/spectra and the coefficient of variation (CV) of the selected peaks' mass was always less than 30% and did not differ statistically between the different sample and laser settings (table 1). The mass accuracy was achieved by external calibration. Using the ClinProt platform described herein, we analyzed 74 serum samples including 30 from SCLC patients and 44 healthy individuals. The discriminatory masses were clustered and selected after noise filtering. These peaks were ranked according to the *p *value of Wilcoxon rank sum test. A total of 100 distinct peptide peaks were unambiguously resolved ranging from 800 to 10 000 Da between two groups. We detected 9 peptides that showed statistically significant differences (*P *< 0.000001). Both top two peaks at 1778.67 and 1865.79 Da were highly expressed in SCLC patients, but weakly expressed in healthy individuals (Figure 1, Figure 2).

**Table 1:** Serum pool (n = 13) in the mass range 1000-10 000 Da, obtained by MALDI-TOF MS after magnetic bead preparation.

Mass	CV
2461.24	0.239559
2981.16	0.245415
3066.44	0.176466
3363.13	0.158828
3613.66	0.162182
4061.46	0.261492
4126.42	0.241348
5019.26	0.187579
5571.19	0.287579
5738.96	0.304054
6646.44	0.174836
7148.49	0.247252

### Peptide Ion Signatures Provide Predictive Model for a Validation Set

All detected peaks were used with a k-nearest neighbor genetic algorithm in ClinProt system to generate a cross-validated classification model. With the highest predictive value, the model comprised five potential biomarkers with m/z of 4172.21, 1211.27, 5336.83, 6333.6 and 7469.23 Da, respectively. While the peaks with m/z 4172.21 and 6333.60 Da, were expressed higher in healthy controls than in SCLC patients, the other three biomarkers appeared to be expressed in an opposite way. The model has a sensitivity of 90.00% (27/30) and a specificity of 97.73% (43/44). 88.89% (8/9) of stage I/II patients were accurately assigned to SCLC group. The descriptive statistics of these five peaks were shown in Table 2.

**Table 2:** The statistics of five candidate biomarkers for screening SCLC(S) from healthy individuals(H)

m/z	p	intensity in S (mean ± SD)	intensity in H (mean ± SD)
4172.21	< 0.000001	37.06 ± 25.13	96.66 ± 33.83
6333.6	0.000523	17.14 ± 7.86	24.97 ± 6.99
1211.27	0.0538	7.28 ± 3.8	5.05 ± 3.91
5336.83	0.0548	389.28 ± 341.28	230.51 ± 99.69
7469.23	0.444	16.28 ± 8.88	14.55 ± 3.72

## Discussion

SCLC is a highly aggressive and chemoresponsive disease in which the best predictor of outcome appears to be staged at diagnosis [4]. This tumor is characterized by a rapid doubling time, high growth fraction, and the early development of wide-spread metastases. Currently, no satisfactory biomarkers are available to screen for small-cell lung cancer (SCLC).

Protein expression profiling of body fluids from patients with cancer has recently become a valuable tool for obtaining information on the state of protein circuits inside tumor cells and outside the cells at the host-tumor interface. During last years, it has been demonstrated that the serum is a convenient protein-rich information reservoir that may show a systemic response to a specific disease [5]. In serum, low molecular weight proteins and peptides that are related to this altered microenvironmental "cancerous" state can be detected.

MS instrumentation and analysis tools have continued to rapidly evolve and improve our ability to detect less-abundant serum proteins. Until now, the most commonly used instrument was the SELDI-TOF MS. This technology has been used successfully to discover potential serum diagnostic markers for breast [6], lung [7], bladder [8], liver [9], and gastric [10]cancers. After the original highly intriguing report that the serum proteome profile can be used for the early detection of ovarian cancer, many researchers have applied the SELDI-TOF MS technology to detect proteome profiles specific for other forms of cancer and non-malignant disease. However, SELDI-TOF MS does not allow a direct identification of the discriminatory proteins and the debate about the reproducibility has been particularly strong [11]. The new ClinProt system providing the optimal reproducibility is suitable for automated protein profiling and has the capability to simultaneously identify potential biomarker proteins. The improved sensitivity and resolution allowed detection of 400 polypeptides (0.8-15 kDa range) in a single droplet (10-50 μL) of serum, and almost 2000 unique peptides in larger sample sets, which can then be analyzed using common microarray data analysis software. It has been successfully used to identify highly sensitive and specific potential biomarkers for the diagnosis of many cancers, but similar studies of lung cancer have not been reported.

There are many tumor markers detected in the sera of patients with lung cancers. Using an array of biomarkers is one way of acquiring a differentiated cancer diagnosis. At present, this is usually performed by means of RIA or ELISA. The most extensively investigated circulating protein markers include tissue polypeptide specific antigen (TPS), neuron-specific enolase (NSE) and pro-gastrin-releasing peptide (ProGRP). It has reported that the sensitivity, specificity and accuracy of SCLC diagnosis by an indicator of TPS and NSE were 84.4%, 87.8%, 83.6% and 79.3%, 93.7%, 88.3%, respectively, In addition, the level of TPS and NSE in the patients' serum with metastatic SCLC were markedly higher than those in the patients with SCLC without metastasis, increased with the number of metastatic focuses [12]. The presence of SOX Group B and/or ZIC2 AAs are frequently observed in small cell lung carcinoma (SCLC), and were also reported to be indicators of a better prognosis [13].

Here we adopted the ClinProt system to establish a fingerprint pattern including five proteins to distinguish SCLC patients from healthy individuals with a specificity of 97.73% and a sensitivity of 90.00%. 88.89% SCLC patients of the early stage were accurately diagnosed. It is noteworthy that our pattern was helpful for the diagnosis of SCLC with early stage. With the highest predictive value, the top two peaks (1778.67 and 1865.79 Da) were unqualified to selected as the set of potential biomarkers, even though they had the significant confidence. Among the five biomarkers, two were up-regulated in cancer patients, and they may be oncogene proteins. Other three were down-regulated in cancer patients, and they may be tumor suppressor gene proteins. The m/z with 5336.83 Da was lowly expressed in SCLC patients. Freed et al [14]. also found a serum biomarker at 5337.62 Da with down-regulated in head and neck squamous cell cancer (HNSCC) samples by MALDI-TOF MS technology. Therefore, this potential biomarker remains interesting to be further investigated.

## Conclusions

In the current study, we were encouraged to find that the combined model successfully classified all 9 stage I/II SCLC patients from healthy individuals, which suggest that our approach is useful for early SCLC detection. However, the sample size in this study is still limited and a larger number of samples are needed to further test our model. Nonetheless, by establishing serum protein fingerprint patterns through MALDI-TOF MS approach, we have provided a novel highly sensitive and specific method for SCLC diagnosis. This study is an initial proof for a successful evolution of the potentially great use of discriminating protein profiles in the detection of SCLC. Further research is needed to confirm our current findings in larger cohorts of study samples.

## Competing interests

The authors declare that they have no competing interests.

## Authors' contributions

YSY designed the experiments, controlled the overall project. DJ wrote the initial version of the manuscript and participated in data collection and analysis. ZW and ZB supported some instruments. LXL, HSF and BLN participated in the design of the study and reviewed the manuscript. NYD and SWL participated in conception and design. All authors read and approved the final manuscript.
